# Inner Ear Conductive Hearing Loss and Unilateral Pulsatile Tinnitus Associated with a Dural Arteriovenous Fistula: Case Based Review and Analysis of Relationship between Intracranial Vascular Abnormalities and Inner Ear Fluids

**DOI:** 10.1155/2015/817313

**Published:** 2015-11-26

**Authors:** Ettore Cassandro, Claudia Cassandro, Giuliano Sequino, Alfonso Scarpa, Claudio Petrolo, Giuseppe Chiarella

**Affiliations:** ^1^Department of Medicine and Surgery, University of Salerno, 84084 Salerno, Italy; ^2^ENT Department, San Giovanni Battista Hospital, University of Torino, 10124 Torino, Italy; ^3^Department of Experimental and Clinical Medicine, Audiology and Phoniatrics Unit, University of Catanzaro Magna Graecia, 88100 Catanzaro, Italy

## Abstract

While pulsatile tinnitus (PT) and dural arteriovenous fistula (DAVF) are not rarely associated, the finding of a conductive hearing loss (CHL) in this clinical picture is unusual. Starting from a case of CHL and PT, diagnosed to be due to a DAVF, we analyzed relationship between intracranial vascular abnormalities and inner ear fluids. DAVF was treated with endovascular embolization. Following this, there was a dramatic recovery of PT and of CHL, confirming their cause-effect link with DAVF. We critically evaluated the papers reporting this association. This is the first case of CHL associated with PT and DAVF. We describe the most significant experiences and theories reported in literature, with a personal analysis about the possible relationship between vascular intracranial system and labyrinthine fluids. In conclusion, we believe that this association may be a challenge for otolaryngologists. So we suggest to consider the possibility of a DAVF or other AVMs when PT is associated with CHL, without alterations of tympanic membrane and middle ear tests.

## 1. Introduction

Conductive hearing loss (CHL) and pulsatile tinnitus (PT) with normal otoscopic examination and normal systemic findings often offer a diagnostic challenge to the otolaryngologist.

CHL usually indicates external or middle ear pathologies, such as fixation of the ossicular chain (otosclerosis), tympanic membrane perforation, and cholesteatoma. When there is no evidence of middle ear disease, it is mandatory to suspect a lesion in the inner ear. In fact, in the last 15 years, basic sciences and clinical studies have increasingly shown that conductive or mixed hearing loss may depend on inner ear structural anomalies, like superior, posterior, or lateral semicircular canal dehiscence, large vestibular aqueduct syndrome, dehiscence between cochlea and carotid canal, Gusher syndrome, Ménière's disease, Paget disease, and other inner ear malformations that mimic the so-called “third window” mechanism [1].

PT is a clinical condition frequently resulting from altered blood flow or increased blood turbulence near the ear or from idiopathic intracranial hypertension. It is often described as a sound synchronous with the heartbeat. It is, almost always, the result of the sound of nonlaminar blood flow that is transmitted to the inner ear [2]. PT could be due to altered blood flow by a variety of factors: generalized increased blood flow (strenuous exercise, pregnancy, severe anaemia, hyperthyroidism, or thyrotoxicosis), localized increased flow (persistent stapedial artery, vascular tumours of the head and neck, and arteriovenous malformations (AVMs)), turbulent blood flow (atherosclerosis), and altered awareness (secondary to CHL, heightened sensitivity in the auditory pathways) [3]. The most common local causes of PT include atherosclerotic carotid artery disease, dural arteriovenous fistulas (DAVFs) and AVMs, glomus tumours of the jugular foramen and middle ear, fibromuscular dysplasia, tortuous internal carotid artery, jugular bulb abnormalities, transverse-sigmoid sinus stenosis and aneurysms, myoclonic contractions of the tensor veli palatini, Paget disease, otosclerosis, superior semicircular canal dehiscence, and idiopathic intracranial hypertension syndrome [3]. The underlying cause of PT is not always easy to identify but every attempt must be made to arrive at a diagnosis because, in the majority of cases, there is a treatable etiology. Failure to establish a conclusive diagnosis could miss rare, but serious, associated intracranial abnormalities.

DAVF is an abnormal connection of the arterial and venous systems involving one or more dural sinuses and branches of the external, internal, and/or vertebral arteries. DAVFs vary greatly in their size and drainage pattern, which leads to a number of presentations and symptoms, including tinnitus, ocular symptoms (diplopia, chemosis, etc.), cerebral venous congestion, intracranial hypertension, and intracranial hemorrhage [4]. To the best of our knowledge only one case have been reported that includes sensorineural hearing loss [5]. DAVFs comprise 10% to 15% of all intracranial AVMs and, most often, involve the dura surrounding the sigmoid and transverse sinuses [6]. The mechanism of development of an acquired DAVF involves three steps: (1) thrombosis of a dural sinus, which leads to impaired venous drainage and increased dural sinus pressure; (2) subsequent dilation of physiologic shunts between the thrombosed sinus and extracranial arteries; and (3) recanalization of the dural sinus, which allows direct arterial shunting into the sinus from the external carotid system [7]. The Cognard classification (Table 1) is one of several methods put forth to correlate the venous drainage pattern with the risk of neurologic complications, if the DAVF is untreated [8, 9]. While type I DAVF has a benign clinical course without the risk for intracranial hemorrhage, types II and III DAVF may have an aggressive clinical course including hemorrhage, neurological deficits, hydrocephalus, dementia, intracranial hypertension in 20% of cases, and hemorrhage in 10%. Intracranial hemorrhage is present in 40% of type III cases and in 65% of type IV. Finally, type V cases, with spinal perimedullary drainage, produce an ascending myelopathy in 50% of cases [8, 10, 11]. The diagnosis of a DAVF relies heavily on the use of radiologic imaging, with conventional cerebral/carotid angiography being the gold standard [4]. Previous studies have shown that magnetic resonance imaging (MRI) as well as magnetic resonance angiography (MRA) may be useful in the assessment of DAVF [12–14]. MRA may be suggestive of a DAVF but frequently is not conclusive, because a normal exam alone cannot rule out a lesion [15]. Treatments used for DAVFs range from noninvasive methods (e.g., self-compression of the fistula) to endovascular embolization, self-expanding stents deployed by balloon angiography [16], stereotactic radiosurgery, or craniotomy [17, 18].

Here we report a rare case where a patient presented with CHL and PT that was diagnosed to be due to a DAVF. Moreover, we made a systematic review of literature for papers reporting this association. Finally, we analyze and discuss the most significant experiences and theories reported in literature about the possible relationship between vascular intracranial system and labyrinthine fluids.

## 2. Case Presentation

A fifty-five-year-old female came into our department in November 2013, suffering from left PT, synchronous with her heartbeat, and CHL on the same side, from two years, with a sudden onset. General examination was normal. Otoscopy and neck evaluation were normal and lateral rotation of the head to the right or left had no effect on the tinnitus, although a mild digital compression over the internal jugular vein was able to eliminate it. Pure tone audiometry showed left-sided CHL, in the low frequencies tones. Immittance measurement showed bilateral type A tympanograms, with the presence of acoustic reflexes. Due to the fact that the patient reported an improvement in hearing during voluntary mastoid compression, we repeated the pure tone audiometry with a thumb placed on the left mastoid causing compression and, surprisingly, this resulted in a temporary normal hearing.

We also carried out a careful evaluation of tinnitus that included both evaluation by questionnaires for psychometric assessment and hyperacusis, as well as psychoacoustic assessment. Our protocol included (1) a through history with detailed interview; (2) questionnaire for hyperacusis [19] and tinnitus handicap inventory (THI) [20]; and (3) our tinnitus test battery: tinnitus loudness and pitch matching, minimal masking levels (MMLs), and loudness discomfort levels (LDLs) [21]. The result was a monolateral, pulsatile tinnitus, with pitch over 3 kHz. LDLs did not show the presence of loudness sensitivity problems; psychometric test (THI) showed that tinnitus was easily masked by environmental sounds and easily forgotten with activities, but that it interferes with sleep. There were no significant results on other tests.

**Figure 1:**
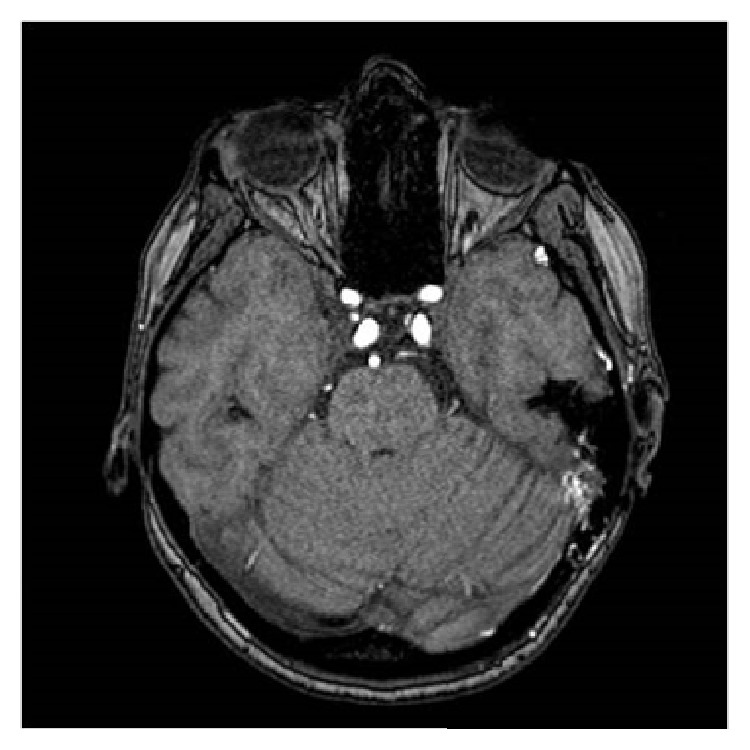
MRI of the brain: the vascular malformation, in the left occipitotemporal region.

**Figure 2:**
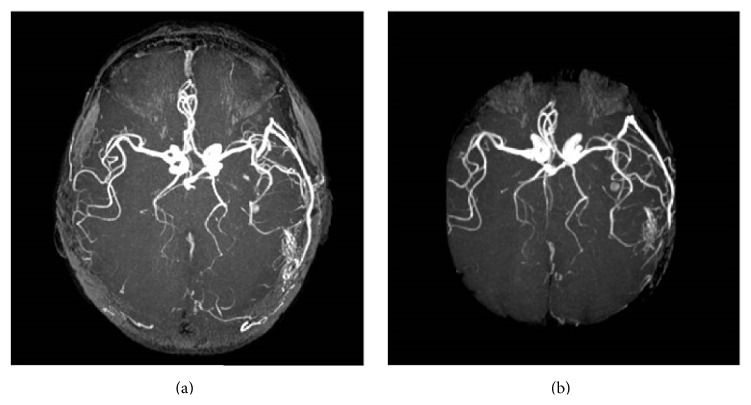
(a) Angiography showed a type II DAVF of the left lateral sinus according to Cognard classification (1995) composed of meningeal branches of middle meningeal artery and of anastomotic branches of left occipital artery. (b) Postintervention angiography.

The patient underwent a MRI of the brain, which detected a vascular malformation in the left occipitotemporal region (Figure 1). Angiography showed a type II DAVF of the left lateral sinus, according to Cognard classification [8], composed of meningeal branches of middle meningeal artery and of anastomotic branches of left occipital artery (Figure 2(a)). The venous drainage was towards sigmoid sinus, left lateral sinus, and petrous sinus, against the stream of flow. The selective injection of the right internal carotid artery showed a 3 cm diameter supraclinoid internal carotid artery aneurysm. At the same time, the patient was taken up for neuroradiological intervention, where the fistula was treated with endovascular embolization wherein all the meningeal branches that fed fistula were occluded using Onyx glue, in the same session. Following this, there was a dramatic disappearance of the tinnitus. Moreover, pure tone audiometry showed a recovery of the CHL on the left side. The follow-up angiography (Figure 2(b)), after three months, showed a regression of the fistula and, at one year, the patient is completely asymptomatic and cured of tinnitus and the hearing threshold was stable.

## 3. Discussion

While the presence of a PT linked to DAVFs is not a rare occurrence [22–24], the association of PT, CHL, and DAVFs is a very rare condition. We made a review of literature, through systematic searches in PubMed (National Center for Biotechnology Information of US National Library of Medicine) by these key words: dural arteriovenous fistula, DAVF, and hearing loss. To our knowledge, only one case has been reported that includes hearing loss associated with DAVF, but, in addition, it was of sensorineural type [5]. All other cases of hearing loss are described as consequences of treatment of DAVFs, whether surgical procedures or embolization [25, 26]. No CHL has been reported to be associated with DAVFs. So we want to analyze briefly the possible relationship between intracranial vascular system and inner ear fluids.

Recent literature suggests that an alteration of venous cerebral circulation, like in consequence of bilateral transverse sinus stenosis (BTSS) [27] or DAVF [22–24], might be the underlying mechanism of PT, independently of presence or absence of elevated values of intracranial pressure [27]. Vascular abnormalities (i.e., BTSS and DAVF) could alter the frail balance between intracranial and labyrinthine fluids with consequences on labyrinth hydromechanics, causing otologic symptoms [28–30]. Previous studies described how intracranial and labyrinthine fluids are connected. The cochlear aqueduct (CA) put in direct relationship the perilymph with intracranial fluids. On the other hand, the endolymphatic sac put in communication, not directly, the endolymph with the intracranial fluids; the pressure balance between cochlear fluids (i.e., endo- and perilymph) is maintained by Reissner's membrane and other labyrinthine membranes [31]. Moreover, out of physiological condition, as in presence of elevated intracranial pressure or anatomical lesions, other connections are represented by the perivascular and perineural spaces in inner ear. The counter-evidence of the connections between the intracranial and labyrinthine fluids comes from previous studies that described that, in animal models, when the cochlear and vestibular aqueducts were closed there are no modifications within inner ear fluids [32, 33].

On this issue, the function of the CA is very intriguing. It seems to perform the role of a low pass filter, preventing diffusion to the inner ear fluids of dangerous variations of intracranial pressure. This CA activity can suffer, on the other hand, a potential loss of efficacy, because of its progressive, interindividual variable and age related degree of closure [31].

To conclude, the dynamics of cerebrospinal fluid (CF), its reabsorption, transportation in the bloodstream, and pressure regulation occurs primarily at the level of arachnoid granulations, placed, to a large amount, in transverse sinuses. Then, it is easy to assume that a venous malformation, like transverse sinus stenosis, could act negatively on CF reabsorption with potential hangover on inner ear fluids dynamics.

In our case, there is a DAVF. So the question is why DAVF generated a low frequencies CHL?

It is known that a CHL may be due to not only external or middle ear diseases. In 1980, Bess et al. [34] reported three cases of unexplained CHL, in which surgery failed to find any plausible explanation for the air-bone gaps. In their conclusion, inner ear fluids anomaly were speculated to be the cause, with no further evidence provided. In the last 15 years, basic science and clinical studies have increasingly shown that CHL can be associated with inner ear anomalies, like semicircular canal dehiscence and enlarged vestibular aqueduct, that are two well-defined clinical entities, called “third window” lesions by some authors [35, 36], in which hearing loss and air-bone gaps can be often found. Muchnik et al., in 1989, described low-frequency air-bone gaps, without middle ear pathology, in 13 of 40 patients with classic Meniere's disease and suggested that air-bone gaps were caused by endolymphatic hydrops and labyrinthine hypertension, which could produce pathophysiological changes in the inner ear fluid dynamics [37].

In our case, we suppose that type II DAVF produced a “subclinical” intracranial hypertension with abnormal venous drainage, with prolongation of parenchymal phase of venous outflow. This mechanism may be responsible for disturbance in the microcirculation of the stria vascularis, resulting in PT and CHL. To clarify this assumption a theory from Godlowski is helpful [38]. He hypothesized that an elevation of the hydrostatic head pressure at the arterial end of the microcirculation in stria vascularis, in case of endolymphatic hydrops, will increase the force which drives fluids from the capillaries into the endolymphatic space. In such an event, the hydrostatic pressure within the endolymph will rise only if the excess fluid is not eliminated at an equal rate, back into the blood at the venous end of the stria vascularis. The venous drainage of the inner ear is carried out by the cochlear aqueduct vein [39] and the vestibular aqueduct vein. The venous blood empties either directly into the inferior and superior petrosal sinus or into internal jugular vein [40].

Based upon both the anatomy of venous inner ear drainage and the pathogenic mechanism suggested by Godlowski [38], an existing excess of endolymphatic volume could be secondary to a chronic reduced or altered venous drainage of the anterior and posterior vestibular veins and/or of the cochlear veins into the venous cerebrospinal system.

## 4. Conclusion

While PT and DAVFs are not rarely associated, the finding of a CHL in this clinical picture is really unusual. This association may be a challenge for most otolaryngologists. On the basis of relationship between intracranial and labyrinthine fluids that we try to describe from the evidences reported in literature, we suggest to consider the possibility of a DAVF or other AVMs when PT is associated with CHL, without apparent alterations of tympanic membrane and middle ear tests. Moreover, it must be borne in mind that DAVF of the left lateral sinus is commonly associated with PT that disappears or decreases in intensity through a simple retroauricular or neck compression, as in the case of our patient. It is also important to rule out idiopathic intracranial hypertension possibility that, in 55%, 60% of cases occur often with a unilateral PT. MRA is the investigation of choice and treatment is directed at closing the arteriovenous fistula or treating the intracranial hypertension.

## Figures and Tables

**Table 1:** Cognard classification of dural arteriovenous fistulas (1995).

Cognard type	Venous drainage pattern
I	Venous drainage into dural venous sinus with antegrade flow
IIa	Venous drainage into dural venous sinus with retrograde flow
IIb	Venous drainage into dural venous sinus with antegrade flow and CVR
IIa + b	Venous drainage into dural venous sinus with retrograde flow and CVR
III	Venous drainage directly into subarachnoid veins (CVR only)
IV	Type III with venous ectasias of the draining subarachnoid veins
V	Venous drainage into spinal perimedullary veins

CVR = cortical venous reflux.
